# Stop vitamins: Low levels of ascorbic acid regulate the transition from cell proliferation to differentiation in Arabidopsis tapetum

**DOI:** 10.1093/plcell/koad047

**Published:** 2023-02-17

**Authors:** Nicolas M Doll

**Affiliations:** Assistant Features Editor, The Plant Cell, American Society of Plant Biologists, USA; Department of Plant Biotechnology and Bioinformatics, Ghent University, Ghent 9052, Belgium; VIB Center of Plant Systems Biology, Ghent 9052, Belgium

Sexual reproduction of flowering plants relies on the successful dissemination of male gametes to female flowers by pollen grains. Pollen grains develop within anthers and are surrounded by an innermost somatic layer of secretory cells, called the tapetum. Tapetal cells provide the nutrients and cell wall components necessary for pollen maturation. Tapetum development follows 3 main phases: first, cell specification and mitosis, then cell differentiation, and lastly programmed cell death (Sanders et al. 1999).

Early development of the tapetum is controlled by several molecular actors, including a network of transcription factors that are well characterized in Arabidopsis (*Arabidopsis thaliana*; Lou et al. 2018). One central element of this network is the MYB transcription factor DEFECTIVE IN TAPETAL DEVELOPMENT AND FUNCTION 1 (TDF1). *tdf1* mutants display supernumerary and poorly differentiated tapetal cells, indicating that TDF1 promotes the transition from mitosis to differentiation. However, how TDF1 regulates this transition remains largely unknown. In this issue, **Si-Yuan Wu, Ling-Yi Hou, and colleagues (Wu et al. 2023)** uncovered a link between TDF1 and vitamin C metabolism that plays a key role in tapetum development.

Vitamin C, also known as L-ascorbic acid or AsA, is the major anti-oxidant buffer in plant apoplasts. AsA is widely used as a reducing agent by diverse apoplastic enzymes. The major biosynthetic pathway for AsA involves the VITAMIN C DEFECTIVE 1 (VTC1) enzyme, which catalyzes the rate-limiting reaction of the pathway. AsA levels in the apoplast are further limited by its oxidation, as oxidized AsA can no longer act as a reducing agent, and by its recycling (Pignocchi and Foyer 2003).

Wu et al. (2023) identified *SKS18* as a direct target gene of TDF1. *SKS18* is specifically expressed in the tapetum and encodes an ascorbic acid oxidase, which oxidizes AsA in the presence of a copper cofactor in vitro. Interestingly, *sks18* mutants display supernumerary tapetal cells, which is reminiscent of the *tdf1* phenotype, though to a weaker extent. As expected, inflorescences of both *sks18* and *tdf1* mutants have higher AsA levels compared to wild-type plants. To test whether these high AsA levels cause an impaired transition from mitosis to differentiation, the authors overexpressed VTC1 in the tapetum and obtained plants with artificially high levels of AsA. They again observed supernumerary tapetum cells, validating the conclusion that a low level of AsA promotes the exit from the mitotic phase.

**Figure 1.**
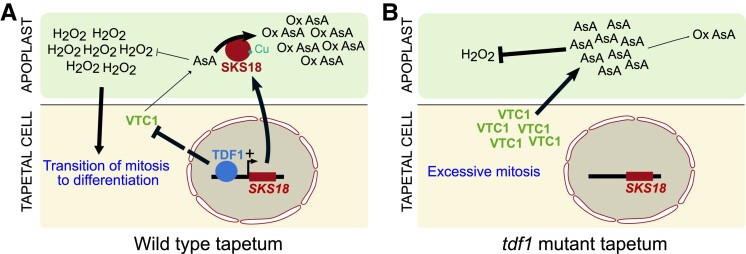
TDF1 promotes tapetal cell differentiation through a reduction in AsA and an increase in H_2_O_2_. **A)** In a wild-type tapetum, TDF1 directly activates *SKS18*, which encodes an apoplastic AsA oxidase. TDF1 also indirectly inhibits the accumulation of the AsA biosynthesis enzyme, VTC1. Consequently, AsA levels drop, leading to higher levels of H_2_O_2_ that promote the transition from mitosis to differentiation. **B)** In the absence of TDF1, AsA accumulates and H_2_O_2_ levels are low, impairing the proper transition from mitosis to differentiation. Figure created by N. Doll with Inkscape.

However, overexpressing SKS18 in *tdf1* did not fully lower AsA concentration to wild-type levels, suggesting additional defects in AsA metabolism. Interestingly, the AsA biosynthesis protein VTC1 accumulates more in a *tdf1* tapetum than in the wild-type. As TDF1 is a transcriptional activator, this accumulation is likely an indirect consequence of the absence of TDF1, which could have impact on VTC1 stability. In summary, TDF1 lowers AsA concentration in the apoplast by reducing AsA biosynthesis and by promoting AsA oxidation through SKS18. A low level of AsA then promotes the transition from mitosis to differentiation (Fig. 1).

The authors investigated the consequences of low AsA on the levels of reactive oxygen species (ROS), as AsA is a strong ROS buffer (Pignocchi and Foyer 2003). As expected, genotypes with high AsA levels also have low hydrogen peroxide (H_2_O_2_) levels in the tapetum. Overexpression of ROS scavengers in the tapetum leads to a similar decrease in H_2_O_2_ and to the appearance of supernumerary tapetum cells, which indicates that the transition from mitosis to differentiation is promoted by high H_2_O_2_.

In conclusion, in the tapetum, the exit from the mitotic phase and the entry into the differentiation phase is regulated by a high concentration of H_2_O_2_ that is a consequence of TDF1 action. Indeed, TDF1 reduces AsA levels by promoting AsA oxidation and by inhibiting AsA biosynthesis. Consequently, the efficiency of the AsA buffer decreases and H_2_O_2_ accumulates, triggering the onset of tapetum cell differentiation (Fig. 1). This work shows a new contribution of ROS to tapetal development, as ROS promotes also the last developmental phase of programmed cell death (Xie et al. 2014). Further studies on how AsA regulates ROS levels during tapetal cell death might be interesting to develop a more complete view of the contribution of AsA to the development of the tapetum.
